# Technical Potential of Biogas Technology to Substitute Traditional Fuel Sources and Chemical Fertilizers and Mitigate Greenhouse Gas Emissions: The Case of Arba-Minch Area, South Ethiopia

**DOI:** 10.1155/2022/6388511

**Published:** 2022-02-04

**Authors:** Tariku Tekle, Getachew Sime

**Affiliations:** ^1^Department of Biology, Hawassa University, Hawassa, Ethiopia; ^2^Center for Ethiopian Rift Valley Studies, Hawassa University, Hawassa, Ethiopia

## Abstract

A study was conducted in South Ethiopia with the aim of assessing the technical potential of biogas energy in replacing traditional bioenergy and chemical fertilizers and mitigating greenhouse gas emissions. A household survey with both a quantitative and qualitative approach was employed for data collection. Primary data were gathered from 182 biogas adopters as well as 10 key informants and three group discussions. Secondary data were also collected from different sources. The average biogas production potential of installed biogas plants was 205 m^3^ per day. The average reduction in use of firewood, charcoal, dung cakes, and crop residues due to biogas adoption was 66%, 72%, 68%, and 89%, respectively. The use of bio-slurry as an organic fertilizer reduced the quantity of chemical fertilizers used by more than 50% per household per year. The reduction in the quantity of biofuel consumption reduced the volume of greenhouse gas emissions by 418 tons of carbon dioxide equivalents per household per year. If the reduced firewood and charcoal use reduced the felling of live trees, this could potentially conserve 45 ha of forest per household per year. Therefore, biogas energy could help reduce the anthropogenic pressure on forest resources by addressing the major drivers of deforestation and forest degradation.

## 1. Introduction

The growing energy demand and negative impacts of fossil fuels on the environment contribute towards the use of biogas as a clean and renewable energy source. Biogas, a methane-rich gas, contains 18.6–26.04 MJ/m^3^ of energy. It is produced by anaerobic fermentation of organic wastes. It is distinct from other renewable energy sources, such as solar, wind, thermal, and hydropower, in that it also controls and collects organic wastes that if untreated could cause severe public health and environmental pollution [1]. Organic wastes are the major inputs for biogas production. Biogas production from such sources offers alternative fuel, biofertilizer, electricity, waste recycling, greenhouse gas emission reduction, and environmental protection [2]. Production of biogas through anaerobic digestion of the organic fraction of animal waste yields between 40% and 70% methane, with the remainder being carbon dioxide, hydrogen sulfide, and other trace gases [3]. The technology allows controlled management of large amounts of animal dung and the safe production of gas for cooking, lighting, or power generation [4].

Ethiopia is a developing country with a large population size. The annual average population growth of the country is nearly 3%. Currently, the total population size is over 100 million, and this number is expected to double in the coming 25 years. The vast majority of the population lives in rural areas where modern energy services are rarely available. Over 92% of this population uses biomass-based energy for cooking [5]. An increasing fraction of the population is facing a difficult choice between eating cooked food and travelling long distances to collect fuel for cooking. As a result, fuelwood is overharvested in many areas, with an accelerating deforestation rate in already ecologically sensitive and vulnerable areas. Furthermore, as deforestation increases, fuelwood and charcoal become increasingly scarce and expensive. Therefore, households and large institutions cope by substituting fuelwood with dung cakes and agricultural residues [6]. The scarcity of fuelwood has led to an increased use of dung and agricultural residues for cooking, which could otherwise have been used to enhance soil fertility and agricultural production [7]. Only a small proportion of rural households have adopted biogas technology across the country in general and in Arba-Minch Zuria District in particular, where the majority of households have persistently continued to use the traditional, inefficient, and unsustainable biomass-based energy systems.

The transition from these traditional biomass fuels to more modern, clean, and efficient energy systems will enhance benefits to a vast number of people and the environment. Understanding the underlying forces affecting energy transition is therefore crucial. One of the means to reduce dependence on traditional use of biomass energy is to promote and supply energy-efficient technologies [8]. Biogas energy offers an attractive option to replace unsustainable use of wood, charcoal, and crop residues as well as to mitigate greenhouse gas emissions. Biogas technology uses locally available resources of organic wastes and water. It is a renewable energy source that addresses the basic energy needs of rural households. It supports decentralized access to household energy. In addition, the bio-slurry by-product of biogas production enhances agricultural productivity and reduces the use of chemical fertilizers. In Ethiopia, the large number of livestock could play an important role in providing dung as a primary feedstock, but experience in biogas systems in Ethiopia is limited. Animal and human excreta are generally available within rural areas, and there is a potential for a vast biogas technology dissemination program for cooking, lighting, and bio-slurry. Moreover, with the continued loss of vegetation in Ethiopia, the country has reached a point at which greater effort is required to diversify energy sources, improve efficiency, and take climate change into consideration in energy planning and development.

In Ethiopia, despite an appreciation of biogas technology as a good option, it has never been adopted to the level needed to reverse the continued energy security crisis and high rate of deforestation. There are a few studies on biogas technology adoption and its realized benefits in various parts of the country [6, 9–13]. Most of these studies report on institutional roles and socioeconomic benefits of the technology. They have not specifically targeted the technical potential of biogas technology in replacing biomass energy sources and chemical fertilizers and mitigating greenhouse gas emission. Therefore, this study was initiated to evaluate the technical potential of biogas in Arba-Minch Zuria District in South Ethiopia. The following are the main questions addressed by this study: What is the exploitable technical potential of biogas for replacing traditional biomass energy sources in the study areas? What is the traditional biomass resource consumption that can be reduced by biogas adoption? What is the estimated greenhouse gas emission reduction potential associated with biogas adoption? What amount of chemical fertilizers could be replaced by the use of bio-slurry and what are the monetary benefits of this?

## 2. Materials and Methods

### 2.1. Description of the Study Area

**Figure 1:**
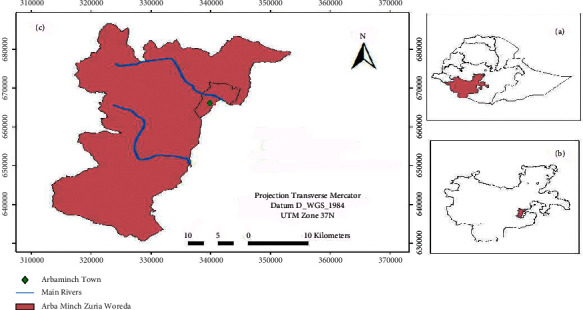
Location map of the study area: (a) South Ethiopia, (b) Arba-Minch Zuria District in South Ethiopia, and (c) Arba-Minch Zuria District.

Arba-Minch Zuria District is one of the districts found in Gamo-Gofa Zone, South Ethiopia. The district is located at a distance of 275 km from the regional city, Hawassa, and 505 km from the country capital, Addis Ababa. The district covers 1001 km^2^ and has 29 rural *kebeles* (A “kebele” is the smallest administrative unit in an Ethiopian administrative structure.) and one district town (Figure 1). It is bordered on the south by the Dirashe special district, on the west by Bonke, on the north by Dita and Chencha, on the northeast by Mirab Abaya District, on the east by the Oromia National Regional State, and on the southeast by the Amaro special district. Part of Gamo-Gofa Zone is located in the Great Rift Valley of Ethiopia that includes Lake Abaya and Lake Chamo and their islands. Nech-sar National Park is also located between these lakes.

Based on the 2007 census conducted by the Central Statistical Authority (CSA), this district has a total population of 164,529 (82,199 men and 82,330 women) [14]. The town of Arba-Minch has a total population of 74,843 (39,192 men and 53,651 women). The population density of the study area varies from 172 people per km^2^ to 226 people per km^2^ [14].

Fifteen years' climatic data (1999 to 2014), particularly temperature and rainfall, from the National Meteorological Services Agency (NMSA) showed that the average monthly minimum and maximum temperature of the study area ranges between 16°C and 37°C. The mean monthly maximum temperature of the study area ranges between 28.1°C in July and 33.8°C in February, while the mean monthly minimum temperature ranges between 15.3°C in December and 18.2°C in April. The general elevation of the district ranges from 1200 to 3300 m above sea level, and the district is characterized by an average annual rainfall of 963.3 mm [15]. The rainfall ranges from 26.45 mm in February to 164.6 mm in April.

The study area has an undulating topography that favors the existence of different climatic conditions. The study area involves six major types of land use or land cover. These are residence, farm land, water bodies, forest, bush lands, and bare lands. The land use system is dominated by farm land that accounts for 46% of the total area. The second dominant land cover type is bush land (34.1%), comprising short trees and shrubs. Settlement areas, dense forest, water bodies, and bare lands account for 0.85%, 0.85%, 12.5%, and 5.7%, respectively. The increasing high urbanization, population growth, and illegal commercial trade of charcoal have negatively affected the forest resources. The main form of income of households is mixed crop-livestock farming.

### 2.2. Study Design

A household cross-sectional survey study was conducted to assess the potential of biogas technology to substitute traditional bioenergy and chemical fertilizers and mitigate greenhouse gas emissions.

### 2.3. Target Population as a Source of Data

Biogas technology adopter households in the study district were the target population for this study. In addition, the District Health Office, Agricultural and Rural Development Office, healthcare workers, and other knowledgeable individuals were purposively selected.

### 2.4. Sample Size Determination and Sampling Procedure

#### 2.4.1. Sample Size Determination

The total number of biogas user households in the district was 300 (Table 1). The list of these households was obtained from the Arba-Minch Zuria District Water, Irrigation, and Alternative Energy Office. The sample size (**n**) is determined using the following formula [16]:

(1) $$\begin{array}{c} n=\frac{NZ^{2}p\left(1-p\right)}{d^{2}\left(N-1\right)+Z^{2}p\left(1-p\right)}, \end{array}$$

where *N* is the total number of households in the locality using biogas energy sources at household level, which is 300; *d* is the error term or degree of precision, which is equal to 6%; *Z* is the distribution level, which is equal 2.58 at 99% of confidence level; and *p* is the proportion of population, which is 50%. Therefore, out of the total population of 300 biogas technology adopter households in the study sites, a sample size of 182 households was surveyed.

#### 2.4.2. Sampling Procedure

The study involved systematic random sampling and purposive sampling techniques. Probability sampling was used to select the sample biogas adopter households, while purposive sampling was used to select key informants and focus group discussants [17]. A sample size of 182 sample households was selected using systematic random sampling from a sampling frame comprising a list of 300 biogas adopters (Table 1). Based on the list of households, the target households were chosen based on (*N*/*n*)^t^ equal intervals, where *n* is the current sample size and *N* is the total biogas adopters households size in the district (*N*/*n* = 300/182 = 1.65), summarized in Table 1.

### 2.5. Data Collection Procedure

Data were collected using a pretested structured questionnaire, key informant interviews (KIIs), focus group discussions (FGDs), and field observations. The data collected through the structured questionnaire were administered by the investigator and data collectors. The structured questionnaire was designed to collect information related to the study of technical biogas production potential, including number of livestock, domestic energy use, forest cover, and deforestation rate from the 182 biogas adopter households. Semistructured questions were used to collect data from energy experts and focus group discussants. Ten experts were interviewed from the Gamo-Gofa Zone Water, Irrigation, and Energy Department and Arba-Minch Zuria District Water, Irrigation, and Energy Office, Agricultural and Rural Development Office, Environmental Protection and Forest Development Office, Livestock Extension Office, Omo-Micro Finance Institution, and GIS Office. These are key stakeholders used in this study as data sources and beneficiaries. Local communities, NGOs, researchers, and other interested individuals working on traditional biomass resource management, forest management, and greenhouse gas emission mitigation were also engaged. Furthermore, a total of 12 individuals were selected as group discussants from village leaders, agriculture development agents, model farmers, women, and individuals with better acceptance at community levels. The composition of the discussants was grouped by age, sex, wealth, and agricultural experience. The most important data collected included availability of a constant supply of manure, number of livestock owned, availability of water for diluting cow dung, suitability of existing temperature and availability of space for installation and bio-slurry disposal, consumption patterns of traditional biomass energy, and bio-slurry use. The field observations were carried out first using an observational checklist followed by a self-administered questionnaire. The checklist was used to assess the actual practice of biogas digester operation, livestock management, water availability, and bio-slurry management.

### 2.6. Technically Exploitable Potential of Biogas Resources

The technical potential of biogas provides an estimate of the total, technically feasible local capacity for biogas production. In-depth structural interviews were carried out to determine the technically exploitable potential resources.

### 2.7. Estimation of the Potential for the Generation of Biogas Energy from Cattle Manure

The number of cattle owned by households was used to estimate the daily dung production. This is because biogas is only produced from cattle manure in the study areas. The amount of feedstock used per day was weighed using a spring balance, which was used to estimate the average dung production per animal per day. The cattle were partly stall-fed and partly free-grazed. The average numbers of adults and young cattle in the study area were considered. Following White et al. [18] and Bond and Templeton [19], estimation of the potential for the generation of biogas from cattle manure is calculated using the following formula:

(2) $$\begin{array}{c} BPt=NHt\times DM\;t\times Et, \end{array}$$

where BP*t* is the theoretical biogas potential (m^3^ CH_4_ per day); NH*t* is the number of livestock, which is given by the average number of cattle per household multiplied by the number of households; DM*t* is the dry weight of manure produced (kg per animal per day), which is 10 kg per day; and Et is the coefficient to convert a given amount of feedstock slurry (dry manure from cattle) into biogas, which is assumed to be 0.0320 m^3^ CH_4_ per kg of dung (ranging from 0.023 to 0.04 m^3^/kg) [20].

### 2.8. Technical Potential of Biogas Technology in Replacing Traditional Fuel Quantity and Type

The amount and type of energy used for cooking in rural areas depend on income, availability of fuel, cooking behavior, and efficiency of appliances [21]. In-depth interviews were carried out to determine fuel demand, expense, and type. Furthermore, in order to minimize over- and underestimation of the traditional biomass fuel consumption, bundles and sacks were first weighed by using a spring balance and then converted into kg. Adopter households were asked to quantify the amount of firewood, charcoal, crop residue, and dung cakes they used on weekly basis, both preadoption and postadoption of biogas technology. Traditional fuel composition was recorded on the basis of mass of backloads and cartloads obtained from the mass of bundles and sacks, collection time, and number of loads collected per day. Although the majority of households use small amounts of electricity for lighting, it was not possible to measure this. This is because most of the households could not recall the actual number of units consumed and paper bills were not available.

### 2.9. Estimation of Greenhouse Gas Emission Reduction

It was assumed that 1 kg of fuelwood generates 1.518 kg carbon dioxide equivalents [22]. Greenhouse gas emission reduction was obtained as follows:

(3) $$\begin{array}{c} {\text{CO}}_{2}\text{ emission reduction}=\text{amount of }{\text{CO}}_{2}\text{ stored in }1\text{ kg of wood}\times \text{amount of }{\text{CO}}_{2}\text{ emission by burning of }1\text{ kg of wood.} \end{array}$$

### 2.10. Determination of Impact on Forest Area and Chemical Fertilizer Use

It was assumed that 1 biogas plant saves 0.3 ha of forest land from deforestation [23]; 1 kg of charcoal requires 5.45 kg of wood [24]; and 712 kg of dry wood is equivalent to 6 large trees [25]. In the study areas, forests are commonly cleared for charcoal making and firewood. Accordingly, a biogas plant saves the amount of trees cleared for charcoal making and firewood through providing an alternative energy sources. Both charcoal and firewood are used for domestic energy, for cooking and lighting. The firewood considered in this calculation is the one obtained through direct cutting down of individual trees. Charcoal is also made from cut trees from forests. Other forest resources like fallen leaves and trees and barks were not considered in this study.

In-depth structural interviews were carried out with respondents to determine the quantity of chemical fertilizer used and the cost to the household. The difference in chemical fertilizer consumption between preadoption and postinstallation for the adopter households gave the amount of chemical fertilizer saved because of the use of bio-slurry.

### 2.11. Data Analysis

Data were entered, coded, and cleaned using Microsoft Excel 2010 and then exported to SPSS, version 20, for further analysis. Data were analyzed using descriptive statistics for most variables using statistical parameters such as mean, minimum, maximum, standard division, frequency, percentage, and cross-tabulation.

## 3. Results and Discussion

### 3.1. Technically Exploitable Potential of Biogas Resources

Out of the 182 installed biogas plants, about 82% were functional at the time of data collection. Cattle dung was the main substrate for biogas production. In addition, about 78% of the households used human excreta as a supplementary substrate. Nevertheless, it was difficult to measure the amount of fecal material per person per day in the survey area due to sociocultural reasons and unavailability of supporting literature in Ethiopia. The average cow dung production per cattle was 8.6 kg per day for partly stall-fed and partly open-grazed in the study area. The total dung production from the functional biogas plants was estimated to be 6417 kg per day. Thus, the potential of biogas production on average was estimated to be 205.02 m^3^ per day, which could be used to cook food for 1108 individuals at the rate of 1.13 m^3^ per day.

Table 2 presents the technical potential resources for biogas production, including the number of livestock per households, the availability of water, and the average dung production per day. The daily production of cattle dung was on average 8.6 kg per day (ranging from 4.0 to 11.5 kg per day). The potential for biogas production calculated from the available cattle manure was 205 m^3^ per day or 74,883 m^3^ per year (ranging from 68 to 80^3^ per year). Potential sources of manure include cattle, small ruminants, and domestic nonruminants, with cattle constituting the majority of the livestock and cattle dung being the major source of feedstock used for generating biogas energy and bio-slurry. Therefore, only cattle manure was used to estimate the biogas production rate. There is a large potential for poultry to provide feedstock for biogas production. From cattle manure alone, the 182 installed biogas plants would provide sufficient biogas energy for 1108 individuals, which is equivalent to 182 households.

The average number of cattle owned by biogas user households was 5, which meets the standard set by EREDPC [7] for installing bio-digesters. The number of cattle owned is a useful indicator of the availability of feedstock for biogas plants. The potential of other feedstock in the study area, such as crop residues or household wastes, has not been fully explored. The cattle management system in the study area is predominantly free-grazing, although some households practice stall-feeding with open-grazing. On-site observations suggested that most farm households had a sufficient supply of cow dung to operate a biogas plant. However, there is difficulty in collecting cow dung from fields because the livestock are mostly free-grazing. Most households were not interested in collecting cow dung from the fields.

Shortage of fodder in both quality and quantity was the major constraint affecting livestock management and daily dung production, so limiting the amount of biogas energy generated for daily consumption. Agricultural residues were a major source of fodder for livestock. For a biogas plant to be attractive to a household, it should be able to provide at least 0.8 to 1 m^3^ biogas energy daily [26]. To generate this amount of biogas, the household should have 20 to 30 kg of fresh dung available on a daily basis [26]. An African household would need at least 3 or 4 night-stabled cattle to achieve this [26]. This requirement is met by a large percentage of households, especially in East Africa [27]. The SNV-Ethiopia indicated that a biogas adopter household should have at least 4 cattle, stabled during the night to be eligible for biogas installation. This could deliver at least 20 kg of fresh dung per day.

The availability of water is another critical factor for feedstock preparation. On average, for biogas adopters, water was available within a 30-minute walk of the household. Eshete et al. [6] also recommended that the source of water should be within a walking distance of 20 to 30 minutes from the home for daily feeding of the biogas digester. As the distance to water increases, the willingness of households to install a biogas digester decreases [12]. Therefore, the technical potential for biogas in relation to availability of water resources in the study area is high. Distance to water sources was a determinant factor in the adoption of biogas technology in Tigray in Northern Ethiopia [9].

### 3.2. Use of Biogas

The biogas was used for making coffee (65.9%), for sauce (58.2%), and for lighting (52.7%). Only 24.7% of the adopter households used biogas energy for baking local bread and none for baking the traditional and staple food, locally known as *Injera* (The staple food in Ethiopia is Injera. A large (60 cm diameter) spongy pancake made of fermented teff dough.) (Table 3).

### 3.3. Reduction of Household Traditional Biomass Fuel Consumption by Use of Biogas Energy

Table 4 presents the diverse range of fuels used by biogas adopters for cooking and lighting. The difference in the amount of traditional fuel consumption before and after installation is the amount of fuel saved as a result of the use of biogas energy (Table 4). Before biogas installation showed that approximately 95.6% of the households used firewood, 68.7% used charcoal and 5.5% used dung cake for cooking and baking *Injera* and local bread. None of the households relied on only one type of fuel. Combination of different fuel types or switching to different fuel sources or types was the strategy used by adopter households to satisfy their energy demands. Firewood was the most consumed and preferred fuel type in the study site. Regardless of stove type, the average firewood consumption per week per household was 44 kg before installation and 15.21 kg after installation of the biogas plant. Similarly, the average dung cake consumption per week per household was 19.86 kg before installation and 6.3 kg after installation of the biogas plant. Adoption reduced the consumption of firewood by 66%, charcoal by 72%, dung cakes by 68%, and crop residues by 89%. This is equivalent to 6.23 kg of firewood for different household activities according to the standard set by EREDPC [7]. The adopter households used on average 2.173 kg fuelwood and 0.9 kg dung cake per day per household for baking *Injera* and local bread and for other cooking purposes. There was a marked reduction in fuel consumption by type and quantity after the installation of biogas plants. However, for baking *Injera* and local bread, fuelwood and charcoal continued to be the two most dominant biomass fuel sources used by the households regardless of stove type. This is attributed to the inefficiency of existing biogas stove in enabling baking of *Injera* and local bread. The continued use of fuelwood and charcoal for domestic energy purposes might be due to the dissemination of energy-efficient and improved stoves by governmental and nongovernmental organizations. These stoves are mainly used for baking *Injera* and local bread even after adoption. At the same time, the adoption of the technology has decreased the use of dung cakes and crop residues for cooking.

### 3.4. Forest Resource Conservation and Greenhouse Gas Emission Reduction in CO_2_ Equivalent

On average, households saved about 2898 kg of traditional fuels per households per year by using biogas energy. Substituting or supplementing charcoal with biogas energy enabled the saving of 0.6256 metric tons of charcoal per household per year (Table 4). Taking into consideration the total biogas users, it was possible to conserve 44.7 ha forest area from deforestation. Furthermore, 3465.8 kg dry wood that would have gone into making charcoal was saved per household per year. This is equivalent to saving 13 trees per household per year (Table 4), which in turn is equivalent to saving 1795 ETB to be incurred for purchasing firewood. This shows the enormous potential of biogas technology in minimizing the anthropogenic pressure on forest resources. The dissemination of biogas technology in the study district, thus, has soundly halted deforestation rate and has contributed more to sustainable management of forest resources. According to Simur [28], biogas technology adoption enabled saving of 2154 kg of fuelwood in Northern Ethiopia, which is equivalent to saving of 0.36 ha of forest per year. Yet, field observation, focus group discussion, and key informant interviews indicated that illegal commercial charcoal and firewood trade have continued to cause vast woody biomass harvest and collection in the study area.

The technical greenhouse gas emission reduction (estimated from the amount of fuelwood reduced by the use of biogas energy) potential and its equivalent carbon emission reduction potential were 1143.783 kg CO_2_eqv per day and 417,768 kg CO_2_eqv per year, respectively. This technical potential was estimated to mitigate about 417.768 tons of CO_2_eqv per year. This will in turn mitigate more amount of GHG emission from deforestation. The biogas sector could, therefore, increase Ethiopia's efforts of further decreasing the already low potential of greenhouse gas emission. The main sources of greenhouse gas emissions in Ethiopia are agriculture, forestry, and energy and industrial sectors [29].

### 3.5. Saving Cost for Purchasing Chemical Fertilizer

The use of biogas technology contributed to the livelihoods of the households through reducing the expenses for purchasing chemical fertilizer. The use of bio-slurry reduced the quantity of chemical fertilizer used and hence households' expense for purchasing them. Before installation of biogas plant, the average amount of chemical fertilizers used per household per year was 117 kg per year. The expenditure on fertilizers per year was 1003 ETB per household. Adoption of biogas reduced the average amount of chemical fertilizer consumption to 56 kg per household per season (Table 5). The use of bio-slurry has, therefore, substituted 61 kg of chemical fertilizer per household per year. In monetary terms, this is equivalent to a saving of 609 ETB per year. This demonstrates that adoption of the technology could greatly reduce the need for chemical fertilizers and, hence, reduce households' annual expenditure.

## 4. Conclusion and Recommendation

There is a large technical potential for biogas technology adoption in Arba-Minch Zuria District in South Ethiopia. Adopter household's livestock size conforms to the minimum requirement set by the national biogas program, which is four heads of cattle. Access to water also meets this standard where it is reachable in less than 30 minutes' walking distance. On average, the biogas technology enabled savings of firewood, charcoal, dung cakes, and crop residues of 1.51, 0.63, 0.71, and 0.05 tons per year per household, respectively. The use of biogas technology has significantly reduced the quantity of fuelwood and charcoal consumption in adopter households. It has replaced 275,209 kg of firewood and 113,863 kg of charcoal per year. In addition, the adoption of biogas technology mitigated greenhouse gas emissions by 417.768 tons of CO_2_eqv and saved 44.7 ha of forest trees per year. These all show the large technical potential of using biogas systems in managing traditional biomass resources and greenhouse gas emissions. The biogas energy use pattern was making coffee, making sauce (*wot*), and lighting that constitute 65.9%, 58.2%, and 52.7%, respectively. Only 24.7% of the adopter households used biogas energy for baking local bread and none for baking the traditional and staple food *Injera*. The existing biogas stove has low energy efficiency. Hence, a large quantity of fuelwood is still used for baking *Injera* and local bread in inefficient traditional stoves. The inefficiency of the biogas stoves becomes one of the major barriers constraining the complete switching of biomass energy to biogas energy systems. Sound actions are needed to enhance the successful exploitation of the potential of biogas systems to meet the renewable energy goals and increase agricultural productivity. The use of biogas technology could significantly reduce the heavy dependence on traditional fuel sources and thereby increase forest coverage and reduce greenhouse gas emissions sourcing from forest sectors. Other feedstock sources should be exploited to increase the technical potential of biogas technology.

## Figures and Tables

**Table 1:** Distribution of sample households and their family sizes.

No.	Rural *kebele*	Total number of user households	Sample user households	Total family size of sample user households
1	Arba-Minch town	16	11	80
2	Kola Shara	65	38	243
3	Kola Shele	46	32	168
4	Shele Mile	13	6	35
5	Chanochalaba	25	15	89
6	Chanodorga	11	7	45
7	Chano Mile	68	48	300
8	Linta	42	24	141
9	Zigiti March	5	-	-
10	Zigiti Eiligo	5	-	-
11	Zigiti Bakole	4	1	7
Total		**300**	**182**	**1108**

**Table 2:** Exploitable biogas potential resources per sample household.

Potential source	No.	Minimum	Maximum	Total	Mean	Std. dev.
Cow and oxen	182	1	15	877	5	2.143
Goats	182	0	15	362	2	2.438
Sheep	39	1	6	103	3	1.328
Donkeys	47	1	6	75	2	1.192
Poultry	182	0	50	1003	6	7.199
Access to water source (time spent for water fetching (minutes per day))	182	1	180	5257	28.9	31.553
Dung production per cattle per day (kg)	182	4.0	11.5	1559	8.6	1.461

Source: own field survey, May 2017, Arba-Minch Zuria District, 2017.

**Table 3:** Major biogas energy use pattern by households.

Biogas energy use pattern	Biogas user households responded “Yes”	%	Biogas user households responded “No”	%
Local bread baking	45	24.7	137	75.3
Lighting	96	52.7	86	47.3
Coffee making and water boiling	120	65.9	62	34.1
Making sauce (*wot*)	106	58.2	76	41.8
Baking *Injera*	—	—	182	100

Source: own field survey, May 2017.

**Table 4:** Preadoption and postadoption biomass energy use pattern by biogas adopter households (HH).

Type of traditional fuel source	Unit of measurement	Average amount fuel used before adoption per HH	Average amount of fuel used after adoption per HH	Mean difference of fuel in kg per HH per week	Amount of fuel saved in ton per HH per year
Firewood (*N* = 182)	kg per week	44.19 kg	15.21 kg	28.98 kg	1.51214 tons
Charcoal (*N* = 182)	kg per week	16.65 kg	4.66 kg	11.99 kg	0.6256 tons
Dung cake (*N* = 182)	kg per week	19.86 kg	6.30 kg	13.56 kg	0.7075 tons
Crop residue (*N* = 182)	kg per week	1.15 kg	0.14 kg	1.01 kg	0.053 tons
**Total fuel composition**	**kg per week**	**81.85**	**26.31**	**55.54**	**2.898**

Assumptions: (i) average weight of 1 bundle of fuelwood was 35.5 kg, and average price of 1 bundle of fuelwood was 66.74 ETB (local market, May 2017); (ii) average weight of 1 sack of quintal charcoal was 30 kg, and average price of 1 sack of a quintal charcoal was 142.93 ETB (local market, May 2017); and (iii) average weight of 1 quintal sack of dung cake was 25 kg, and average price of 1 sack of quintal dung cake was 35.44 ETB (local market, May 2017). 1 US$ = 22.97 ETB (Ethiopian Birr) at the time of data collection. Source: field survey, Trade Transport Office of Town Arba-Minch, May 2017, and National Bank of Ethiopia, May 2017.

**Table 5:** Amount of chemical fertilizer used before and after biogas plant installation.

Amount of average chemical fertilizer used before installation per HH per crop season (kg)	Amount of average chemical fertilizer used after installation per HH per crop season (kg)	Mean difference due to installation per HH per crop season (kg)	%
116.6	55.9	60.7	50.1

**Sources**: field survey, price of 100 kg of chemical fertilizer was 1003 Birr (Arba-Minch Zuria Agricultural and Rural Development Office, May 2017), 1 US$ = 22.9712 ETB (National Bank of Ethiopia, May 2017), and HH = households.

## Data Availability

All data are included within the manuscript in tables and figures.
